# A Case for Diagnosis

**Published:** 1909-03-06

**Authors:** 


### A CASE FOR DIAGNOSIS.

A man, aged fifty-three, a stove-fixer by trade, who had lived temperately in London, and who had hitherto had no occasion to consult a doctor since he was a boy, sought advice on account of a severe pain in the lower part of the right side of the abdomen, in the inner and upper part of his right thigh, and in the right testicle. The pain had come on suddenly three days before, and had increased in severity. It was not equally bad all the time, but it was always present, and there were exacerbations. The pain was hardly less at night when he was in bed than it was during the day, and it greatly interfered with his sleeping. There had been no vomiting.

On examination the patient was found to be fresh complexioned, well nourished and healthy; he did not look at all ill. The pain made it almost impossible for him to walk, so he was lying up on a couch. Nothing abnormal could be seen in the region of the pain; no lump could be felt in the right iliac fossa nor elsewhere; local palpation caused him increased pain. There was no obvious rigidity of the muscles on the right side of the abdomen as compared with those of the left. No abnormal physical signs could be detected in the heart or lungs. Neither liver nor spleen could be felt. There was no jaundice and no evidence of ascites. The urine was carefully examined: it was clear, rather high coloured, of specific gravity 1022, and acid in reaction; no pus, blood corpuscles, renal tube casts, nor abnormal epithelial cells were detected microscopically, though there were small numbers of minute crystals of calcium oxalate. There was no blue line upon the gums. The knee jerks were present, and the pupils reacted to light and to accommodation; the only abnormality to be found in the nervous system was the severe pain apparently along the course of the right genito-crural nerve. The bowels were habitually regular, and rectal examination showed nothing wrong there. The patient's temperature was just 100°F., his pulse rate 84, and respiration rate 16 per minute. The leucocyte count was 8,200 per c.m.

A provisional diagnosis was made, and it proved wrong. The correct explanation of the pain was not even discussed as a possibility at the time. Four days later something happened which cleared up the diagnosis at once. The patient ultimately got perfectly well without any operation.

### Solution.

The provisional diagnosis made was renal colic, probably due to calculus. Repeated examinations of the urine for blood, pus, and crystals were carried out in the expectation of blood at least being present sometimes. Four days after the first consultation a full crop of vesicles appeared in the course of the right genito-crural nerve, and the diagnosis was clearly *herpes zoster*.

